# miR-125b induces cellular senescence in malignant melanoma

**DOI:** 10.1186/1471-5945-14-8

**Published:** 2014-04-24

**Authors:** Anne Marie Nyholm, Catharina M Lerche, Valentina Manfé, Edyta Biskup, Peter Johansen, Niels Morling, Birthe Mørk Thomsen, Martin Glud, Robert Gniadecki

**Affiliations:** 1 Department of Dermatology, Faculty of Health and Medical Sciences, University of Copenhagen, Bispebjerg Hospital, Copenhagen, Denmark; 2 Department of Forensic Medicine, Section of Forensic Genetics, Faculty of Health and Medical Sciences, University of Copenhagen, Copenhagen, Denmark; 3 Department of Pathology, University of Copenhagen, Faculty of Health and Medical Sciences, Bispebjerg Hospital, Copenhagen, Denmark

**Keywords:** hsa-miR-125b, Melanoma, Senescence, In-situ-hybridization, Mel-Juso

## Abstract

**Background:**

Micro RNAs (miRs) have emerged as key regulators during oncogenesis. They have been found to regulate cell proliferation, differentiation, and apoptosis. Mir-125b has been identified as an oncomir in various forms of tumours, but we have previously proposed that miR-125b is a suppressor of lymph node metastasis in cutaneous malignant melanoma. Our goal was therefore to further examine this theory.

**Methods:**

We used in-situ-hybridization to visualise miR-125b expression in primary tumours and in lymph node metastasis. Then using a miRVector plasmid containing a miR-125b-1 insert we transfected melanoma cell line Mel-Juso and then investigated the effect of the presence of a stable overexpression of miR-125b on growth by western blotting, flow cytometry and β-galactosidase staining. The tumourogenicity of the transfected cells was tested using a murine model and the tumours were further examined with in-situ-hybridization.

**Results:**

In primary human tumours and in lymph node metastases increased expression of miR-125b was found in single, large tumour cells with abundant cytoplasm. A stable overexpression of miR-125b in human melanoma cell line Mel-Juso resulted in a G0/G1 cell cycle block and emergence of large cells expressing senescence markers: senescence-associated beta-galactosidase, p21, p27 and p53. Mel-Juso cells overexpressing miR-125b were tumourigenic in mice, but the tumours exhibited higher level of cell senescence and decreased expression of proliferation markers, cyclin D1 and Ki67 than the control tumours.

**Conclusions:**

Our results confirm the theory that miR-125b functions as a tumour supressor in cutaneous malignant melanoma by regulating cellular senescence, which is one of the central mechanisms protecting against the development and progression of malignant melanoma.

## Background

Replicative cellular senescence is the principal mechanism limiting proliferation of normal human cells [1,2]. After approximately 40–60 cell divisions (the Hayflick limit) the cells enter a senescence-associated, irreversible mitotic arrest and exhibit characteristic features comprising flattened morphology with abundant cytoplasm and biochemical markers such as senescence-associated beta-galactosidase (SA-beta-gal), p53 and cell cycle Inhibitors (p16, p21, p27) [3]. Telomere shortening is the primary mechanism of replicative senescence in normal diploid cells [4].

It has recently been discovered that various forms of DNA and cellular damage may cause premature cellular senescence before achieving the Hayflick limit*.* Interestingly, activation of oncogenes provides a potent senescence signal (oncogene-induced-senescence, OIS) which is considered to be an early protective mechanism against development of cancer [3,5-7]. Increased SA-beta-gal staining is seen in a variety of pre-malignant conditions, such as lung adenomas, congenital naevi, benign prostatic hyperplasia and premalignant prostatic intraepithelial neoplasia supporting the role of OIS in early control of malignancy [3,5,8,9]. It has also been observed that progression to the invasive tumour stage is associated with a suppression of OIS [5,6,9-11]. Cellular senescence may also be a mechanism inhibiting cancer progression, since senescence-like state can be induced in established tumours and cancer cell lines by a variety of mechanisms including chemotherapeutic agents or radiation (therapy-induced senescence, TIS) [11,12].

Cutaneous malignant melanoma (MM) is a common, highly aggressive cancer derived from the melanocytes in the skin. MM is an area of high medical need since the metastasis occurs early despite a very low primary tumour mass and metastatic disease is highly resistant to chemotherapy and radiotherapy. MM cells are resistant to apoptosis and the induction of cellular senescence could be explored as a novel approach to therapy [13].

MicroRNAs (miRNA) are 21–25 nt non-coding RNA molecules, which bind to the 3′UTR of mRNAs conferring translational inhibition or degradation [14]. MiRNAs have been found to regulate cell proliferation, differentiation, and apoptosis [15-17] and they have also been implicated in the regulation of senescence (miR-29, miR-30, miR-34a, miR-34b, miR-34c, miR122 miR-203, miR-205 and miR-217) [18-23] mostly by interfering with either the p53 pathway or the retinoblastoma RB1/E2F function. By an extensive analysis and comparison of miRNA expression levels Kozubek et al. showed that it was possible to distinguish melanoma speciments from benign neavi based on the miRNA signature [24]. This supports the theory that miRNA level may influence the development MM.

Two independent research groups have reported a miRNA-dependent induction of senescence in MM cell lines with a focus on miR-205/miR-203-E2F axis [23,25].

By comparing miR expression profiles in metastasizing and non-metastasizing MM we proposed that miR-125b is implicated in the progression of human MM [26]. MiR-125b expression is decreased in the primary cutaneous MM producing sentinel node metastasis comparing to the T-stage-matched non-metastasizing tumours [26]. This has later been confirmed by other groups [27,28]. Mir-125b is a known oncomir and has been implicated in the pathogenesis of leukemias and B-cell lymphomas, breast cancer, squamous cell carcinoma, urothelial carcinoma, prostate carcinoma and colon cancer [29-35]. In MM it has been shown to be related to the pigmentation level [27]. Kappelmann et al. showed that treatment of MM cells with pre-mir-125b resulted in a strong suppression of cellular proliferation [28].

We have gathered preliminary evidence that miR-125b may be involved in the regulation of senescence in MM [36]. In this paper we show that upregulation of miR-125b induces senescence and might constitute one of the possible mechanisms of the suppressive effect of miR-125b in MM.

## Methods

### Cell culture

Human MM cell line Mel-Juso (DSMZ, Braunschweig, Germany) was grown in DMEM Glutamax (Invitrogen, Carlsbad, CA) with 10% FBS (Life Technologies, LT) in 37°C and 5% CO_2_. Transfected Mel-Juso cells were cultured in selection medium, which was the same medium supplemented with 10-μg/mL blasticidin (Invitrogen).

### Plasmid transfection

Mel-Juso cells were transfected with a miRVector plasmid containing a miR-125b-1 [UCSC Genome Bioinformatics: uc010rzr.1] [37] insert and a blasticidin resistance gene (miRVec-125b) [38] or the control miRNA Vector plasmid with a blasticidin resistance gene but without any insert (miRVec-control) (Source BioScience, LifeSciences, Nottingham, UK) (map and sequence of miR-125b, see Additional file 1: Figure S1). 300.000 cells were seeded out 24 h before transfection to reach a confluency of 70–90%. 1 μg of the plasmid was mixed with 400 μl OPTI-MEM (Invitrogen) and 5 μl Lipofectamine RNAiMax (Invitrogen). The mixture was incubated at room temperature (RT) for 20 min and added to freshly washed Mel-Juso cells in 1600 μl culture medium for 24 h followed by a 14-day-culture in the selection medium in 37°C/5% CO_2_. miR-125b expression was confirmed using PCR in each clone. Optimisation in the beginning of the study showed that the transfection efficacy in Mel-Juso cells was higher with Lipofectamine RNAiMax than any other transfection reagents. Transfection procedure was done in four replicates for each plasmid and each evaluated using PCR.

### Flow cytometry

For the measurement of BrdU incorporation, Mel-Juso cells transfected with miRVec-125b or the miRVec-control were pulsed for 20 min with 10 μM BrdU, fixed in 70% ethanol for at least 2 h, washed with phosphate-buffered saline (PBS), incubated in 2 N HCl for 30 min, washed in neutralizing buffer (0.2 M Na_2_B_4_O_7_, pH 8.5) and resuspended in dilution buffer (0.5% Tween 20 and 0.5% bovine serum albumin in PBS) prior to the addition of anti-BrdU mouse antibodies (Becton Dickinson, Franklin Lakes, NJ). Goat anti-mouse antibodies labeled with Alexa-Fluor 488 (1:1000; Invitrogen, Carlsbad, CA) were applied as secondary antibodies. DNA was stained with 0.0003% (30–40 μl per samlpe) 7-amino-actinomycin D solution (7AAD; Beckman Coulter, Brea, CA). Samples were analyzed by means of flow cytometry (Beckman-Coulter, Fullerton, CA). BrdU-positive events (FL1) were plotted versus cellular DNA content (FL3).

### Tumour formation in mice

Mice experiments were performed in accordance with the national law for the experiments on animals of 14 Jun 2011 (full text available on https://www.retsinformation.dk/Forms/r0710.aspx?id=145380) and supervised by an authorised researcher (CML). All mice work is approved by Dyreforsoegstilsynet (generel permit for our laboratory work in mice, permit number 2012-15-2934-00419). All surgery was performed under sodium pentobarbital anesthesia, and every effort was made to minimize suffering.

Six Male BALB/C nude mice (Taconic, Rye, Denmark), 7 weeks of age, were housed in separate boxes with free access to water and standard laboratory food, in an animal facility at a 12 h light/dark cycle and the temperature 23°- 24°C. Mice were acclimatized for one week before the experiments. Subsequently they were anesthetized with 0.05 ml HypDorm given subcutaneously and 5 x 10^6^ Mel-Juso cells transfected with miRVec-125b vector or the control vector in 0.2 ml PBS were injected subcutaneously into the left or right flank, respectively. Tumours with a diameter of at least 1 mm were mapped separately for each animal and followed until these reached a diameter of 12 mm or for a maximum time of 4 weeks. Animals were killed and tumours were weighed and cut into three parts, which were fixed in RNA Later (for RNA isolation), 4% formalin (for standard histology, immunohistochemistry and in situ hybridization) or were frozen at −80°C (for SA-beta-gal staining). Formalin fixed samples were embedded in paraffin and serial sections were stained with hematoxylin and eosin, Ki-67 and cyclin D1, according to standard histopathology protocols. The quantification of Ki-67 and cyclin D1 expression was done semiquantitatively in each tumour using the following ordinal scale: grade 1 (0%-25% positive tumour cells), grade 2 (26%-50% positive cells), grade 3 (51%-75% positive cells), grade 4 (76%-100% positive cells). Lungs and liver were taken out and cut in to three parts each, which were treated as the tumour samples and checked for distance metastases.

### In situ hybridization (ISH) for miR-125b

Formalin-fixed and paraffin-embedded human tissue samples from the archives of the Department of Pathology (Rigshospitalet, Copenhagen, Denmark) were stage T1-T4 cutaneous MM and matched lymph node metastases (14 males and 3 females; mean age 52 years, range 24 to 83 years); mean Breslow depth 1.53 mm (range 0.37 to 4.00 mm); 15 superficial spreading MM and 2 nodular MM. Mouse tumour samples were prepared as described above. Paraffin sections (6 μm) were mounted on SuperFrost®Plus slides (Dako, Glostrup, Denmark) and air-dried for 1–2 h at RT. Sections were deparaffinized in xylene and hydrated through decreasing ethanol concentrations into PBS. Proteinase-K treatment 15 μg/ml in PK-buffer (5 mM Tris–HCl, pH 7.4, 1 mM EDTA, 1 mM NaCl) was performed at 37°C for 8 min in a volume of 300 μL in a Dako hybridizer (Dako). Sections were washed twice in sterile PBS and immediately dehydrated through an increasing gradient of ethanol solutions. Aliquots of 3 different Mercury LNA miRNA Detection Probes; hsa-miR-125b, scrambled probe, and U6 snRNA (Exiqon, Vedbaek, Denmark) were then denatured by heating to 90°C for 4 min and diluted to 40 nM, 40 nM, and 1 nM, respectively, in a formamide-free ISH buffer (Exiqon). 50 μL probe mixture was hybridized with the tissue sections in the hybridizer at 55°C for 60 min. The slides were placed at RT in 5× saline-sodium citrate (SSC) (Invitrogen) and stringency washes were performed for 5 min each at 55°C in 5× SSC (one wash), 1× SSC (two washes) and 0.2× SSC (two washes). The sections were then washed in PBS and blocked with DIG Wash and Block Buffer Set (Roche, Mannheim, Germany). Alkaline phosphatase (AP)-conjugated anti-DIG (Roche) was diluted 1:800 in the blocking solution and incubated for 60 min at RT. Slides were washed twice with PBS containing 0.1% Tween-20. Ready to use tablets (Roche) of 4-nitro-blue tetrazolium chloride (NBT) and 5-brom-4-chloro-3′-indolyl-phosphate (BCIP) substrate were dissolved in aqueous 0.2 mM levamisole. Slides were incubated for 120 min at 30°C to develop the dark-blue NBT-formazan precipitate. Sections were washed twice for 5 min in KTBT buffer (50 mM Tris–HCl, 150 mM NaCl, 10 mM KCl), and then twice in water, dehydrated in the ethanol gradient and mounted.

### SA-beta-gal staining

Monolayers of Mel-Juso cells transfected with miRVec-125b or the miRVec-control after a 24-h culture in 4-chamber glass (Nunc, Rochester, NY) or cryostat tissue sections from mice mounted on SuperFrost®Plus slides were washed twice in PBS and fixed in 0.5% glutaraldehyde (pH = 7.0) for 15 min at RT. The samples were washed twice with 20 ml 1 mM MgCl_2_in PBS (pH = 6.0) and stained with the SA-beta-gal staining solution containing 1 mg/ml of 5-bromo-4-chloro-3-indolyl beta-galactopyranocid, 4% dimethylformamide, 0.0012 mM potassium ferrocyanide and 1 mM MgCl_2_ in PBS (pH = 6.0) for 4 h (Mel-Juso) or overnight (melanocytes) at 37°C [3,5]. Percentage of positive cells was evaluated blindely by one of the investigators (RG).

### Western blotting

Cells were washed in PBS and lysed in the sample buffer 0.5 M Tris–HCl pH 6.8; 5% glycerol; 10% SDS; DTT 0.2 M) supplemented with protease inhibitor cocktail (Roche). Equal amounts of protein were separated by a 4-8% and 12% Bis-Tris gel electrophoresis at 200 V followed by electrophoretic transfer to a nitrocellulose membrane (Bio-Rad Laboratories, Hercules, CA). Membranes were blocked for 1 h at 4°C with Li-Cor blocking agent (Li-Cor, Lincoln, NE) before incubation with the primary antibodies against β-actin (mouse) (Sigma Aldrich, St. Louis, MO), p16 (mouse) (BD Pharmingen, San Diego, CA), p21 (mouse) (Dako), p27 (mouse) (Santa Cruz Biotechnology, Santa Cruz, CA) or p53 (rabbit) (Dako) overnight at 4°C. Subsequently, they were incubated for 1 h with the appropriate secondary antibodies labeled with 800IR dye (anti-rabbit) (Li-Cor), Alexa Fluor 680 (anti-mouse or anti-rat) both from Molecular Probes (Invitrogen). Protein bands were detected and quantified with the infrared Odyssey imaging System (Li-Cor). Quantified intensities were adjusted to the relevant actin intensity and control and sample was then compaired.

### Clonogenic assay

10.000 Mel-Juso cells transfected with miRVec-125b or the miRVec-control were seeded in 10 cm petri dishes containing 10 ml selection medium and cultured for 10–14 days until the appearance of macroscopically visible colonies. The plates were washed, fixed in paraformaldehyde for 24 h and stained with crystal violet 0.05% (Sigma Aldrich) for 20 min before washing in water. Plates were photographed (microscope Olympus IX 70 and camera Nikon D60) and colonies were counted manually.

### Cell viability and apoptosis assessment

1.500.000 Mel-Juso cells transfected with miRVec-125b or the miRVec-control were seeded in 10 cm Petri dishes containing 10 ml selection medium, the medium changed after 24 h and all (floating and adherent) cells collected after another 24 h. Unfixed cells were stained simultaneously with FITC-Annexin-V and PI, according to the manufacturer’s protocol (Beckman-Coulter, Fullerton, CA) and analyzed by means of flow cytometry (Beckman-Coulter, Fullerton, CA) as described previously [39].

### RNA isolation and real-time q-PCR (RT-qPCR)

Mel-Juso cells transfected with miRVec-125b or the miRVec-control were collected and washed twice in PBS. Murine tumour tissue samples were homogenized using a TissueLyzer II (Qiagen, Valencia, CA, USA) and processed as previously described [40]. Small RNAs were purified using the mirVana Isolation Kit (Ambion, Foster City, CA) and the PureLink RNA Micro to Midi Kit (Invitrogen) according to the manufacturer’s instructions. The concentration of RNA was measured spectrophotometrically using NanoDrop ND-1000 (Thermo Scientific, Wilmington, DE), and RNA integrity was confirmed with Agilent 2100 Bioanalyzer using Agilent Nano RNA kit (Agilent Technologies, Santa Clara, CA). miR-125b expression was measured in the samples containing the same amount of RNA with quantitative qRT-PCR assay (TaqMan TM microRNA Reverse Transcription kit-4366596, and TaqMan Universal PCR Master mix-4324018, Applied Biosystems, Foster City, CA) and validated primer sets (Applied Biosystems) according to the manufacturer’s instructions. MiR-191 was used as a reference for the normalization of qRT-PCR-data. The q-PCR was performed in triplicates using a 7900HT Fast Real-Time PCR System (Applied Biosystems).

### Sequencing

DNA was extracted from transfected Mel-Juso cell lines containing either miRVec-125b or miRVec-control using QIAamp DNA mini-Kit (Qiagen) in accordance with the manufacturer’s protocol. PCR was made with reagents from Life Technologies as follows: Master mix (10% Gene amp PCR buffer II, 2.5 mM MgCl_2_, 400 μM dNTP, 0.2 μM of each primer and 1U Ampli Taq gold) was prepared and 7.2 μL was mixed with 16.8 μL of ddH_2_O and 1 μL of 5 ng/μL DNA (template). PCR was setup on an Eppendorf MasterCycler gradient cycler (Hamburg, Germany) with the following program: 95°C 10 min, then 35 cycles of: 95°C 30 s, 62°C 30 s and 72°C 1.5 min; ended with 72°C 10 min and 4°C hold. The PCR product was visually inspected on a 2.2% agarose gel on the Flashgel system (Lonza, Basel, Switzerland). The remaining PCR product was purified on Qiaquick columns (Qiagen) according to manufacturer’s specifications and eluted in 50 μL buffer. Four μL of purified PCR product were used as template in the sequencing reaction.

Sequencing was performed in duplicates with the BigDye V.1.1 cycling sequencing kit (Life Technologies) Samples consisted of 4 μL of Ready reaction mix, 2 μL of BigDye buffer, 3.2 pmol of primer (forward-GCGTTTAAACTTAAGCTTGGTACCGAGC, reverse-CATTCCCCCCTTTTTCTGGAGAC), 4 μL of template and 20 μL of ddH_2_O. Reactions were performed with forward and reverse primers in duplicate with the following program: 96°C 1 min, then 25 cycles of: 96°C 10s, 50°C 5 s and 60°C 4 min, ending with 4°C hold. PCR products were purified with Centrisep columns (Princeton Separations, Adelphia, NJ) according to the manufacturer’s recommendations. Two μL of purified sequencing product were mixed with 8 μL of HiDi formamide and run on an ABI3130xl DNA Sequencer (Life Technologies) with the following specifications: Array 36 cm, polymer POP4, injection voltage 3 kV, injection time 7 s, run voltage 15 kV and run time 1700 sec. One of the reverse duplicates was removed due to low quality. Results were analyzed and assembled with Sequencher v.5 (Gene Codes Corporation, Ann Arbor, MI, USA). Differences in the chromatograms were visually inspected. Low quality regions resulting in differences were removed. Sequence ends had to be cropped due to low reaction quality.

### Statistics

All numeric results are given as mean ± SD unless stated otherwise. *t*-test was used for intergroup comparison. P < 0.05 was considered significant.

## Results

### miR-125b is expressed in human primary cutaneous MM and lymph node metastases

**Figure 1:**
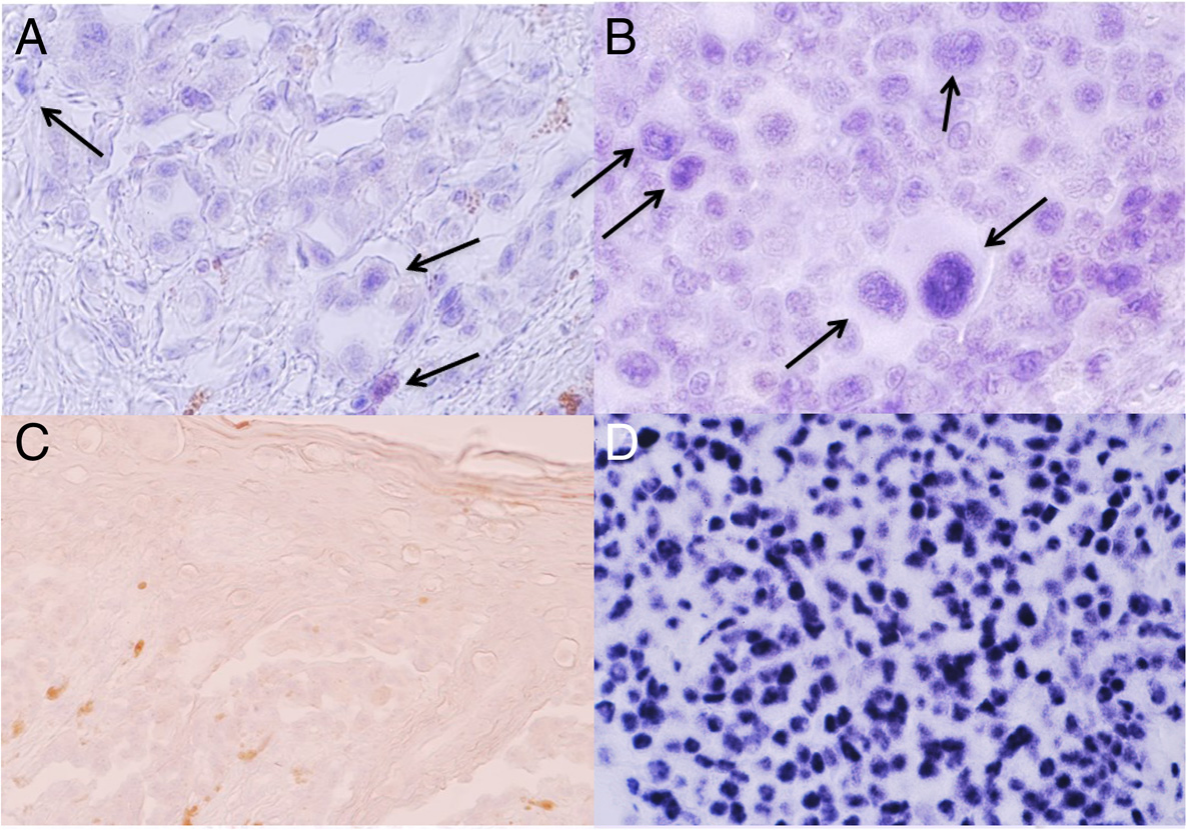
**Expression of miR-125b in primary cutaneous malignant melanoma and lymph node metastases shown with ISH.** Primary cutaneous MM (stage T2; **A**, **C**) and a sentinel node with micrometastases **(B)** were hybridized with the probe for miR-125b **(A, B)** or with the control, scrambled probe **(C)**. Arrows show the cells with prominent staining for miR-125b. **(D)** U6 probe (positive control). Magnification: **A** x60, **B** x40, **C** x20, **D** x20.

We have previously shown by miRNA array approach that miR-125b is expressed in primary cutaneous MM [26,41]. By comparing metastasizing and non-metastasizing stage T2 cutaneous MM we detected an overall decrease in miR-125b expression in metastasizing tumours [26]. Here we employed ISH to further define the expression pattern of miR-125b in MM. As shown in Figure 1 miR-125b is expressed in MM cells both in the primary tumours and in the sentinel node metastases. The staining of miR-125b seems to be located predominantly to the nuclei. We noted that the expression was not homogenous and some cells characterized by large size and abundant cytoplasm expressed higher amounts of miR-125b than other cells (arrows in Figure 1A,B). These larger cells could represent a more malignant, highly atypical tumour cell population, but could also comprise the population of cells undergoing spontaneous cellular senescence. Since SA-beta-gal staining is not possible on paraffin-embedded material and fresh samples from primary cutaneous MM are not available due to ethical considerations, we decided to examine the potential functional involvement of miR-125b in MM cell proliferation and senescence in vitro.

### miR-125b inhibits proliferation and induces senescence in human melanoma line Mel-Juso

Mel-Juso cell line is established from the primary tumour of a 58-year-old woman with MM in 1977. This line has wildtype BRAF, is poorly differentiated [42] and exhibits intermediate invasiveness [43]. Transfection with miRVec-125b resulted in an 8.0 ± 1.13-fold upregulation of miR-125b by RT-qPCR compared to the miRVec-control transfected cells. After the experiments were completed, the miR-125b insert was sequenced to confirm that no mutation had occurred in the miR-125b sequence of the insert throughout the experimental period. For full insert consensus sequence, see Additional file 1: Figure S2.

**Figure 2:**
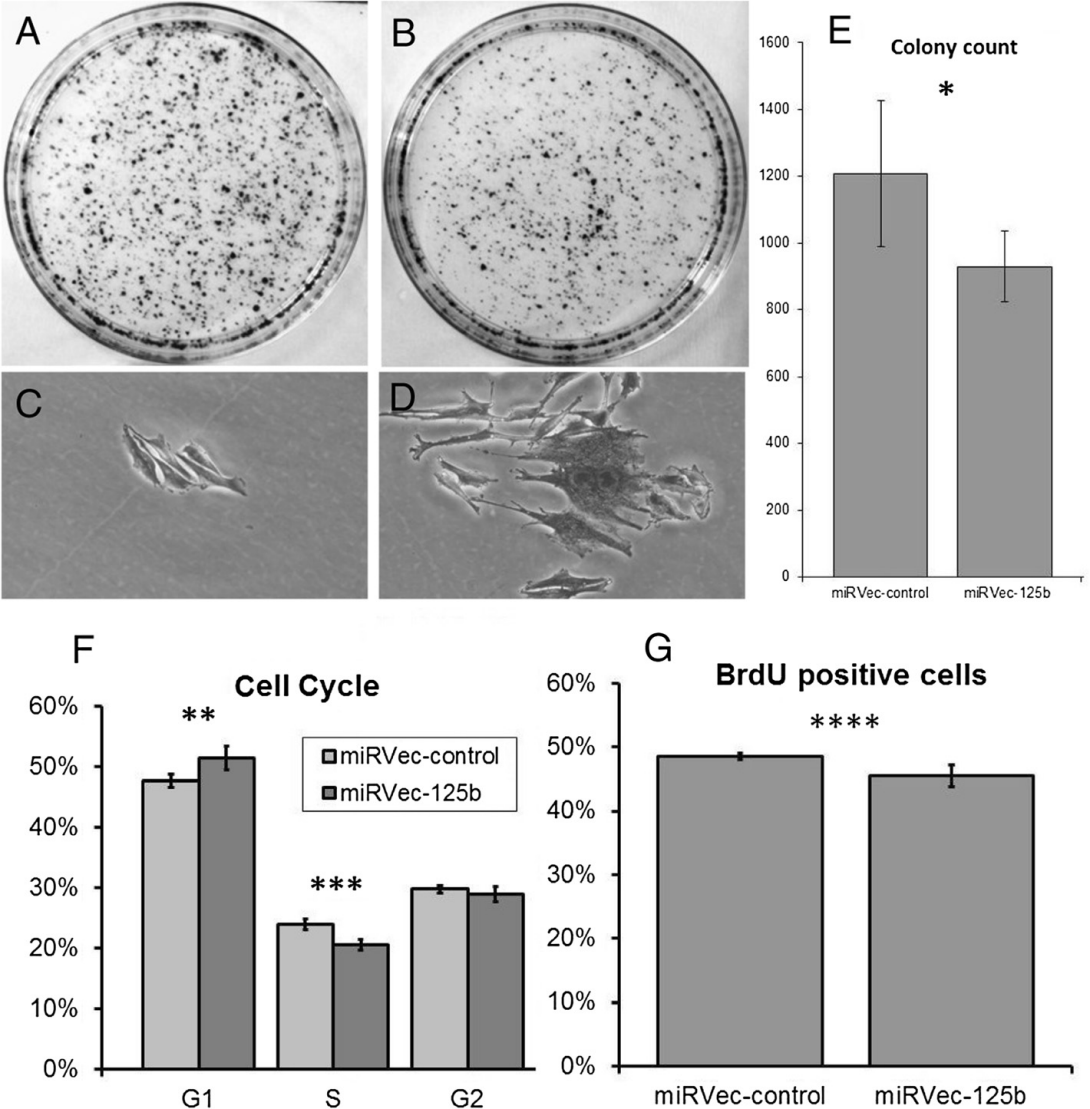
**miR-125b inhibits the proliferation rate in cell line Mel-Juso.** Transfected cells with miRVec-125b vector **(B, D)** or miRVec-control plasmid **(A, C)**. Colony formation **(A, B)**, morphology of the colonies **(C, D)**. **(E)** Quantification of colonies from the colony formation assay. * p = 0,011. **(F)**: Cell cycle analysis. The percentages of cells in G0/G1, S and G2/M phases are plotted for each group. ** p = 0,044, *** p = 0,01 **(G)** Cell proliferation measured by BrdU incorporation **** p = 0.039.

As shown in Figure 2A,B and E the miRVec-125b transfected cells formed smaller and fewer colonies than the control cells (930 ± 105.2, n = 4 replicates vs. 1207.5 ± 218.8, n = 4 replicates, p-value 0.011). The miRVec-125b transfected cells showed a G0/G1 cell cycle arrest, demonstrated in Figure 2 (F, G) by a significantly increased proportion of G0/G1 cells (p < 0.05) and a significant decrease in BrdU incorporation (p < 0.05).

**Figure 3:**
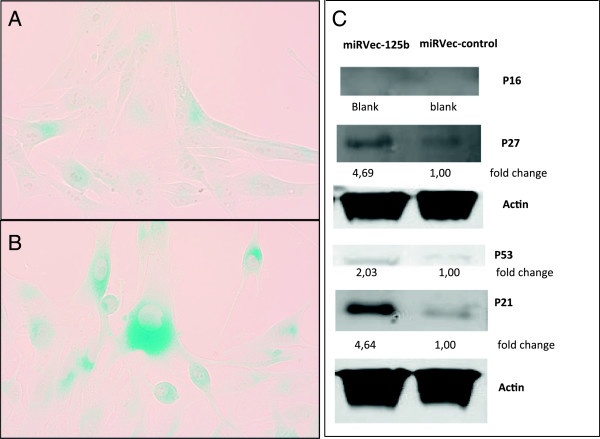
**miR-125b induces senescence in melanoma cell line Mel-J uso.** SA-beta-gal staining for senescence stained in the miRVec-control **(A)** and miRVec-125b **(B)** cells. **(C)** Western blot of senescence markers p16, p21, p27 and p53 and complimentary actin bands.

Microscopic examination of the colonies (Figure 2C,D) revealed that the miRVec-125b-transfected cells were enlarged, flattened, and had abundant cytoplasm, consistent with cellular senescence, in contrast with the normal spindle-shaped cell morphology in the control group. SA-beta-gal staining showed increased expression of SA-beta-gal in estimated 60-70% of the miRVec-125b transfected compared to estimated 5-10% of the control cells (Figure 3A,B). Western blot analysis of the miRVec-125b cells showed an up regulation of the expected markers of senescence: p53, p21 and p27 compaired to the control. p16 was not expressed in the Mel-Juso cell line. (Figure 3C).

To evaluate whether apoptosis was responsible for the apparent lesser growth observed in the colony forming assay we compared the proportions of annexin-positive cells in the miRVec-125b transfected cells with the controls. We found no evidence of apoptosis in either group (95.5% ±0.7% annexin-negative, viable cells in the miRVec-125b transfected cell line versus 94.7% ±1.2% in the control; p = 0.46).

### miR-125b expression in Mel-Juso cells induces senescence and reduces proliferation in a murine tumour model

**Figure 4:**
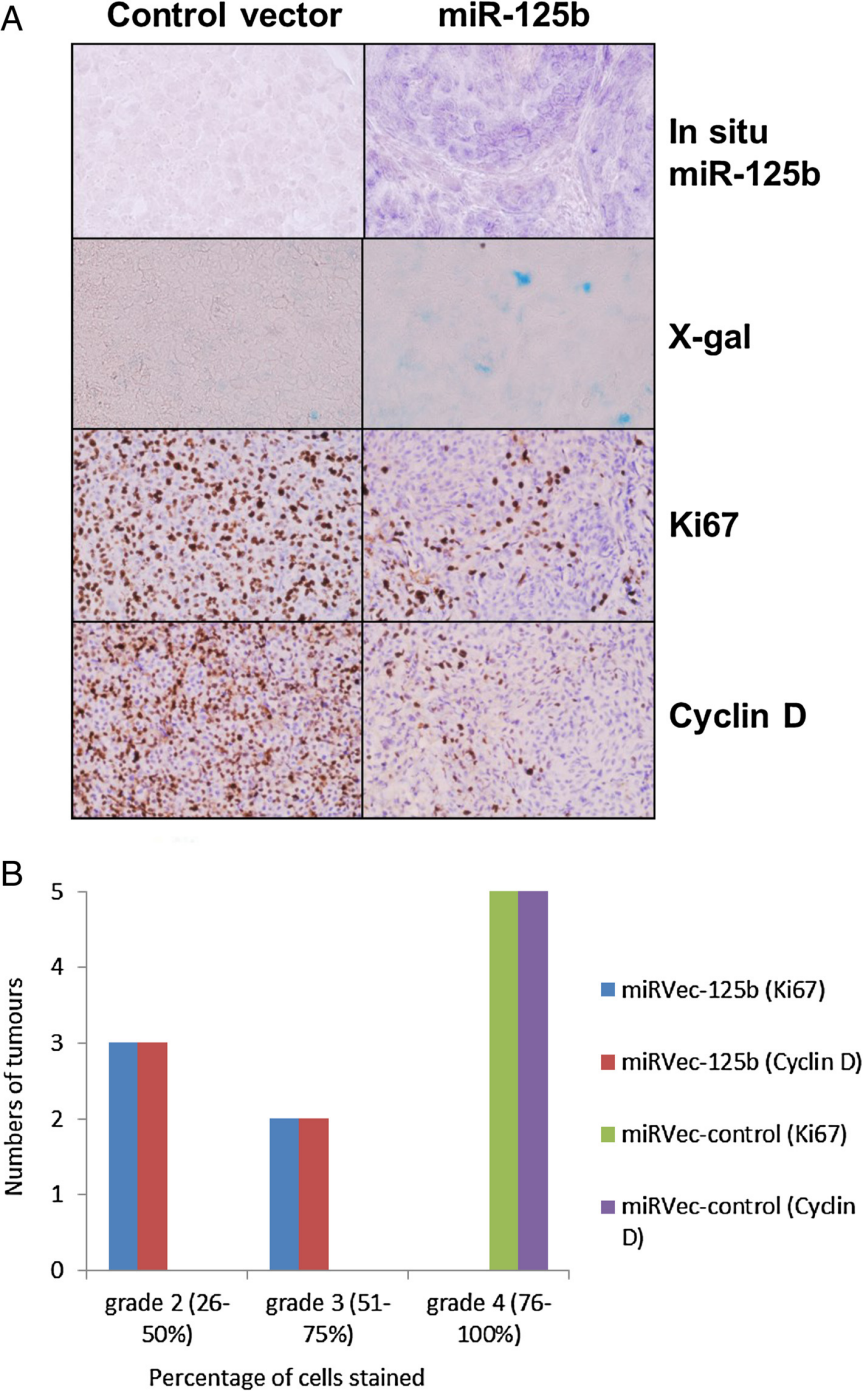
**Reduced mitotic activity and increased senescence in tumours emerging from miRVec-125b transfected Mel-Juso cells shown by (A) ISH with miR-125b (In situ miR-125b), SA-beta-galactosidase staining (X-gal), Ki67 and Cyclin D.** The different stainings were not made in paralelle. **(B)** Quantification of Ki67 and cyclin D expression in murine tumours by visual grading of the proportion of positive cells. In one case the control and experimental tumours were too small for microscopic analysis and therefore the graph shows data for 5 tumours.

To further investigate the significance of miR-125b in the regulation of MM growth we established an in vivo model of tumour formation from Mel-Juso cells injected subcutaneously into immunodeficient, nude BALB/c mice. In this model the Mel-Juso cells formed subcutaneous tumours within a time span of 4 weeks, but did not produce distant metastases (confirmed by macroscopic and histological examination of lung and liver tissue). ISH revealed considerable expression of miR-125b in the tumours originating from the miRVec-125b vector-transfected cells, in contrast to a negligible expression in the tumours emerging from the miRVec-control cells. The tumours were very homogeny in their appearances which was consistent with the fact that they were developed from a clone rather than the normal progression from normal tissue to tumour with the following heterogeneity otherwise known from Melanoma tumours [44,45]. The stability of miR-125b overexpression was confirmed by RT-q-PCR quantification of miR-125b in the tumour tissue showing an 11.6 ± 6.0 fold increased expression in the miRVec-125b transfected tumours. As could be expected from the in vitro experiment, SA-beta-gal staining revealed a focal increase in the staining suggesting accelerated senescence in the tumours originating from miRVec-125b -transfected cells. This was paralleled by the marked decrease in the expression of proliferation markers Ki67 and cyclin D1 (Figure 4). However, we did not detect any difference in weight between the tumours originating from miRVec-125b -transfected cells and the control, miRVec-control transfected cells (mean weight miR-125b: 0.021 g ± 0.003, control: 0.023 g ± 0.004).

## Discussion

This study documented that stable, ectopic expression of miR-125b induced cellular senescence in a MM cell line Mel-Juso, both in vitro and in a xenotransplantation model in mice in vivo. The results were surprising, since miR-125b is generally considered to be an oncogene and in a transgenic mouse model miR-125b overexpression causes myeloid leukemia and B-cell malignancies [46,47]. However, our and other’s preliminary evidence suggested that miR-125b has an anticancer effect in MM [26,28,36,41] and its decreased expression correlated with the metastatic potential of primary cutaneous MM. In the present study we substantiated this hypothesis using a model of stably transfected Mel-Juso cell line with the miRVec-125b vector previously developed by Voorhoeve et al. [38]. We were able to demonstrate an increase in senescence markers (SA-beta-gal, p27, p21, p53) and decreased proliferation with a G0/G1 arrest in miRVec-125b-transfected cells as compared with the control. miRVec-125b -transfected cells were tumourigenic in vivo, but the tumours showed an increased rate of senescence and decreased amount of proliferating cells, as measured by Ki67 and cyclin D1 staining. Together with the results of ISH showing that miR-125b is expressed in primary human MM and lymph node metastases, the data indicate that miR-125b is implicated in the regulation of senescence in human MM.

The mechanism by which miR-125b overexpression promotes cellular senescence is unknown. MiR-125b may negatively regulate the p53 tumour suppressor gene and Bak1 [48] but such a downregulation has not been seen in Mel-Juso cells in our experiments (data not shown). Other known targets are BMF and Lin28a [49] but they are unlikely to be involved in cellular senescence in MM. In general, the functional role of miR-125b seems to be cell specific, since in some types of cancer (e.g. urothelial carcinoma) this miR seems to acts as a tumour suppressor [34].

MiR-dependent induction of cellular senescence in MM has recently and independently been demonstrated by other researchers for miR-203 and miR-205 in cutaneous MM and for miR-34a in uveal MM [23,25,50]. A common biochemical pathway in these cases seems to be mediated by E2F and downregulation of Akt. This is an unlikely mechanism in the case of miR-125b since the screening for the effect of miR-125b overexpression on Akt, Stat, mTOR did not show any changes in the expression level in MM cell lines and E2F2 and E2F3 proteins are not expressed in Mel-Juso cells (data not shown).

In this study we used ISH to evaluate the miR-125b expression locally in the tissue. We observed that the ISH on human tissue samples showed a strong nucleic concentration of miR-125b. This has been surprising since the conventional theory requires the cytoplasmic presence of miRNA for its proper function [51-53]. However, the studies on the compartmentalization of miR-125b have been done with cell lines and not tissue samples, like in our study. On the other hand, our ISH staining on the murine tumours showed a more pronounced cytoplasmic staining. One explanation of this could be that the miR-125b distribution in the cell is different between cells from a cultivated cell line and cells from tissue samples. Kobuzek et al. [24] showed that the miR-125b signal in MM is different in tissue and cell lines. Another explanation could be that the specific distribution varies from tissue to tissue. This is supported by the fact that ISH staining for miR-125b on other tissues have showed that in some tissues there is a clear staining of the nuclei [54-56] while in others the staining is mainly cytoplasmatic [57,58]. This will have to be validated in further studies. It is finally possible, that nuclear miR-125b have a distinct biological role, as previously suggested for other miRNA species.

Another limitation of this study is that the functional role of miR-125b has only been investigated in Mel-Juso cells and it remains to be seen whether the same is valid for other MM cell lines and primary melanocytes. These experiments were attempted but failed due to massive apoptotic response caused by miRVec-125b plasmid. It is known that Mel-Juso cells are very resistant to apoptosis, which enabled us to achieve a stable miR-125b overexpression. Second, we focused primarily on the effect of the sustained overexpression of miR-125b in MM cells. The use of inducible miR-125b vectors will be helpful to elucidate the effect of acute changes in miR-125b levels.

## Conclusion

Our results confirm the theory that miR-125b function as a tumour supressor in cutaneous malignant melanoma by regulating cellular senescence, which is one of the central mechanisms protecting against the development and progression of malignant melanoma.

In view of the recent developments in the use of miRNA mimics and inhibitors for therapy, it is conceivable that miR-125b would be utilized for the treatment of MM by inducing senescence of cancer cells. However, as exemplified by our murine MM model, miR-125b induces senescence focally in the tumours and the effect on tumour mass is negligible. It is known that miRNAs act primarily as switches and amplifiers of the cellular signaling pathways and their physiological effect is rarely very strong [21]. It is therefore our goal for future research to identify which signaling pathways of therapeutic relevance are targeted by miR-125b and devise strategies by which mir-125b overexpression may amplify the effect of anticancer drug.

## Abbreviations

ISH: In situ hybridization; miR-125b: MicroRNA 125b; miRNA: MicroRNA; MM: Malignant melanoma; OIS: Oncogene-induced-senescence; PBS: Phosphate-buffered saline; TIS: Therapy-induced senescence.

## Competing interests

The authors declare that they have no competing interests.

## Authors’ contributions

AMN helped design the study and coordinated the in vitro experiment, made all transfections and western blots, RNA-extractions, participated in the flow studies and the PCR-work, performed the statistical analysis and drafted the manuscript. CML carried out the in vivo studies and the in-situ-hybridizations. VM carried out the PCR-work. EB carried out most flow studies. PJ carries out all sequencing work. NM supervised the sequencing work. BMT processed the tissuesamples for parafine embedment and made the HE, Ki67 and Cyclin D stainings. MG helped with study design. RG designed the study, helped drafting the manuscript and helped with the statistical analysis. All authors read and approved the final manuscript.

## Pre-publication history

The pre-publication history for this paper can be accessed here:

http://www.biomedcentral.com/1471-5945/14/8/prepub

## Supplementary Material

Additional file 1: Figure S1 MiRVec-map. **Figure S2.** Insert consensus sequence. Click here for file
