# Supplementary material for: miR-125b induces cellular senescence in malignant melanoma
Source: BMC Dermatol. 2014 Apr 24;14:8. doi: 10.1186/1471-5945-14-8 (PMC4021480; doi:10.1186/1471-5945-14-8)
Supplement: Additional file 1: Figure S1 — MiRVec-map. Figure S2. Insert consensus sequence. [file 1471-5945-14-8-S1.pdf]

Suplimentary Figure 1.

MiRVec-map.

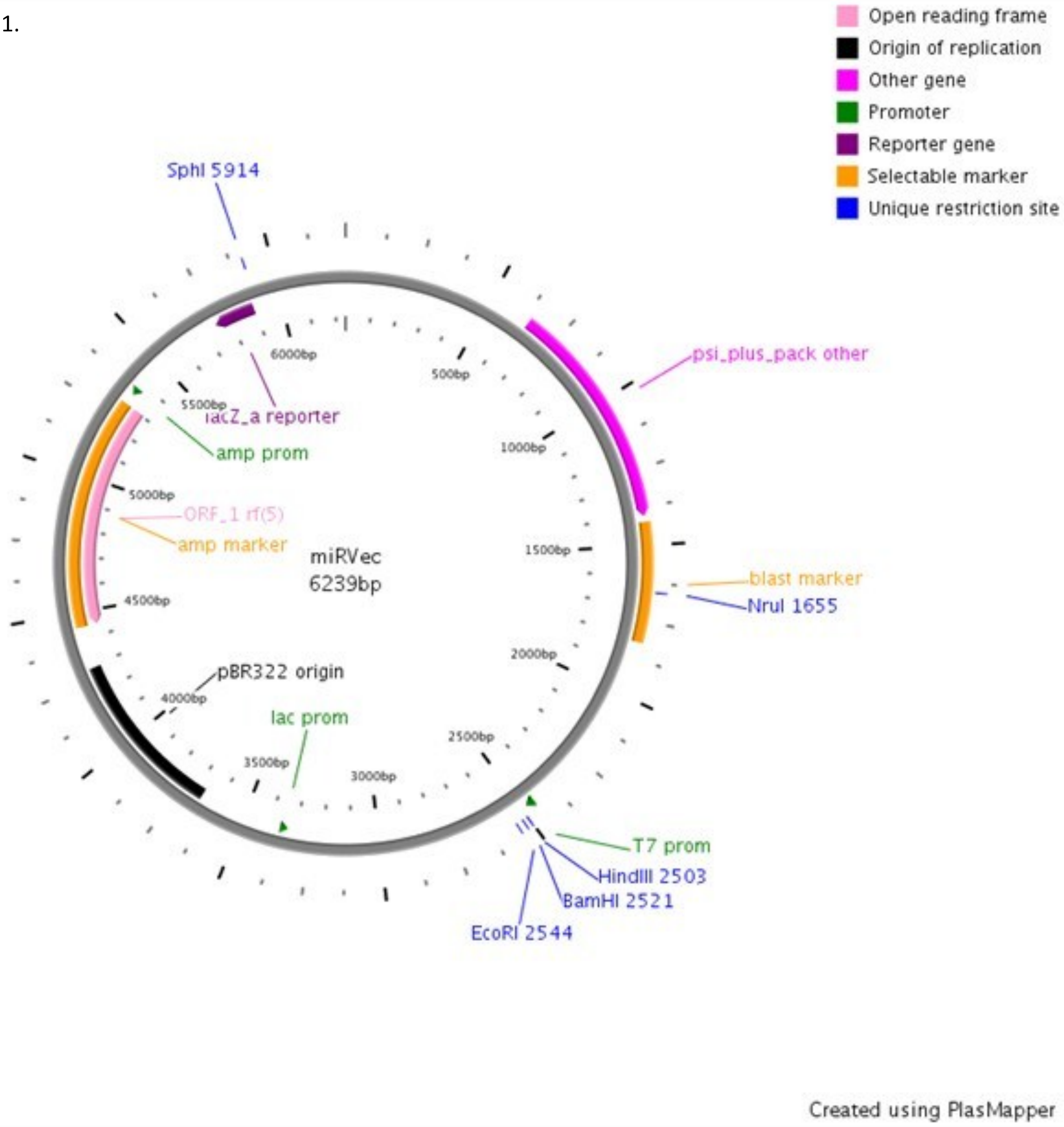

Suplimentary Figure 2.

Insert consensus sequence:

TCGTATGTGTATGTGCGTGATTGTATATGCGCCCCCAGATACTGCGTATG

TGTGTATATATGTATTAGGCTTAAACGGAATCTCAATTTTGTGAAGGAAA

GGAGCTTAGAGAAGAAATACCATACCACCTGTTTGTTGCATCTTAGTTAT

GAACCTCGAACAGAAATTGCCTGTCATTCTTGTTTTGCCTTGCTTTGTCT

CAAGAAAGAAAACATTGTTGCGCTCCTCTCAGTCCCTGAGACCCTAACTT

GTGATGTTTACCGTTTAAATCCACGGGTTAGGCTCTTGGGAGCTGCGAGT

CGTGCTTTTGCATCCTGGAAATTTGGTGGAATTTTATTCTTTAAAGCAAA

AACAAAAGAAAAGAAAGTTTGTCTGAGGTGATTGAGTATACCTCTGAGGT

TTTCATTGTTAGATGGGATCAGGTGACCAGAGAGTGGCAGCTCCTGGATT

TCTGTTAAAGTAGGTATATATTTTGCTTTAATAGCGTGTTGGCTGCTATG

TGTATG
